# RASopathies – what they reveal about RAS/MAPK signaling in skeletal muscle development

**DOI:** 10.1242/dmm.050609

**Published:** 2024-06-07

**Authors:** Katherine A. Rauen, William E. Tidyman

**Affiliations:** ^1^ Department of Pediatrics, Division of Genomic Medicine, University of California Davis, Sacramento, CA, 95817, USA; ^2^ University of California Davis MIND Institute, Sacramento, CA 95817, USA

**Keywords:** Cardio-facio-cutaneous syndrome, Costello syndrome, Myopathy, Neurofibromatosis type 1, Rare disorder, RASopathy, RAS pathway, Skeletal myogenesis, Treatment

## Abstract

RASopathies are rare developmental genetic syndromes caused by germline pathogenic variants in genes that encode components of the RAS/mitogen-activated protein kinase (MAPK) signal transduction pathway. Although the incidence of each RASopathy syndrome is rare, collectively, they represent one of the largest groups of multiple congenital anomaly syndromes and have severe developmental consequences. Here, we review our understanding of how RAS/MAPK dysregulation in RASopathies impacts skeletal muscle development and the importance of RAS/MAPK pathway regulation for embryonic myogenesis. We also discuss the complex interactions of this pathway with other intracellular signaling pathways in the regulation of skeletal muscle development and growth, and the opportunities that RASopathy animal models provide for exploring the use of pathway inhibitors, typically used for cancer treatment, to correct the unique skeletal myopathy caused by the dysregulation of this pathway.

## Teaser

**Summary:** The mechanistic examination of the human condition combined with *in vitro* and *in vivo* modeling studies demonstrate how RAS pathway dysregulation in RASopathies affects mammalian skeletal muscle development.

## Introduction

**Fig. 1.**
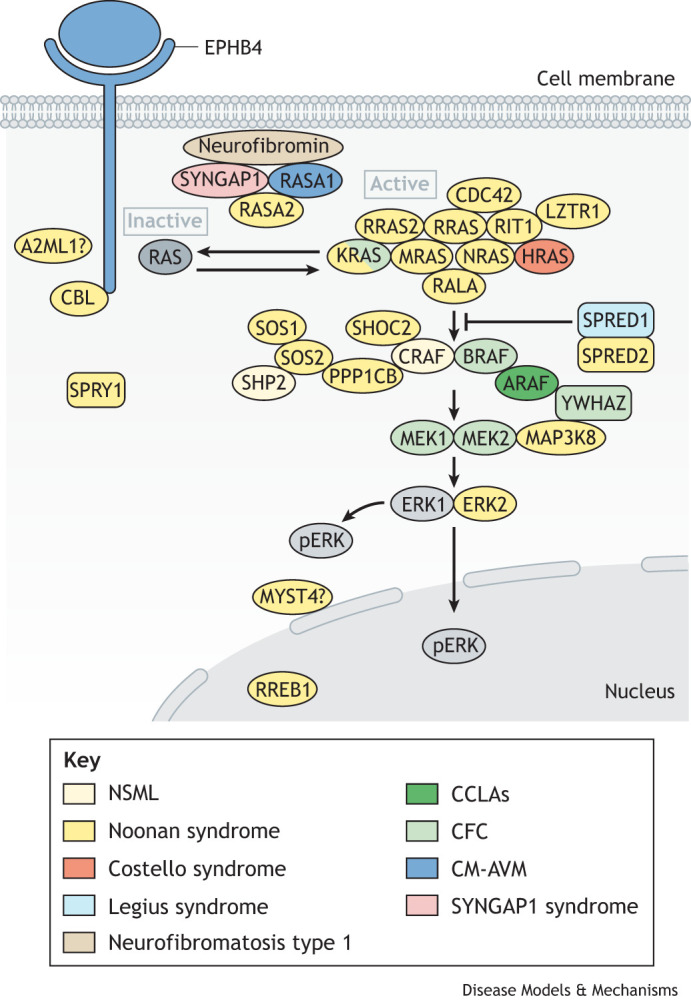
**RASopathies and the affected components of the RAS/MAPK pathway.** The highlighted RASopathies are caused by germline variants in genes that encode components or regulators of the RAS/MAPK signal transduction pathway. These syndromes and their causative genes include neurofibromatosis type 1 (NF1, beige), capillary malformation-arteriovenous malformation syndrome (CM-AVM, blue), Costello syndrome (CS, red), central conducting lymphatic anomalies (CCLAs, green), Legius syndrome (light blue), cardio-facio-cutaneous syndrome (CFC, light green), Noonan syndrome (NS, yellow), Noonan syndrome with multiple lentigines (NSML, light yellow) and SYNGAP1 syndrome (pink). Inactivated RAS is stimulated by numerous environmental stimuli, here depicted by stimulation through the receptor tyrosine kinase EPHB4, which is one of the causes of CM-AVM. There are numerous RAS family members (HRAS, KRAS, MRAS and NRAS) that, once activated, go on to activate RAF family members, i.e. ARAF, BRAF and/or CRAF. These RAFs, in turn, phosphorylate and activate MEK1 and/or MEK2, which then can phosphorylate and activate ERK1 and/or ERK2. Phosphorylated ERKs (pERK1 and pERK2) have numerous nuclear and cytosolic substrates. All causative RASopathy pathogenic variants, whether they cause loss of function or protein activation, result in increased phosphorylation of ERK1 and/or ERK2, the final effectors of the RAS/MAPK pathway. Shown here are all the gene products that have been identified in the literature to cause a RASopathy phenotype, with some pathway components requiring more investigation to identify their pathogenetic and biochemical mechanisms of action. KRAS, depicted in light green and yellow, causes both CFC and NS phenotypes. Specific mutations in the protein tyrosine phosphatase SHP2, the protein product of the gene *PTPN11*, and CRAF (both in light yellow) cause NSML, but specific variants may also cause NS (components in yellow).

RASopathies are rare, single-gene, autosomal dominant (or rarely autosomal recessive) disorders caused by germline pathogenic variants in the genes that encode components of the RAS/mitogen-activated protein kinase (MAPK) signal transduction pathway (Tidyman and Rauen, 2009). The term RASopathy originated in an effort to describe a phenotypically similar group of medical genetic syndromes that include neurofibromatosis type 1 (NF1, the first known genetic syndrome associated with the RAS/MAPK pathway), Noonan syndrome (NS), Noonan syndrome with multiple lentigines (NSML), Legius syndrome, Costello syndrome (CS), cardio-facio-cutaneous syndrome (CFC), capillary malformation-arteriovenous malformation syndrome (CM-AVM), SYNGAP1 syndrome and central conducting lymphatic anomalies (CCLAs) (Rauen, 2013) (see Table 1 for an overview of the key clinical characteristics of these RASopathies). Although each syndrome individually might be considered rare, collectively, RASopathies represent one of the largest groups of multiple congenital anomaly syndromes known (Fig. 1).

**Table 1.**
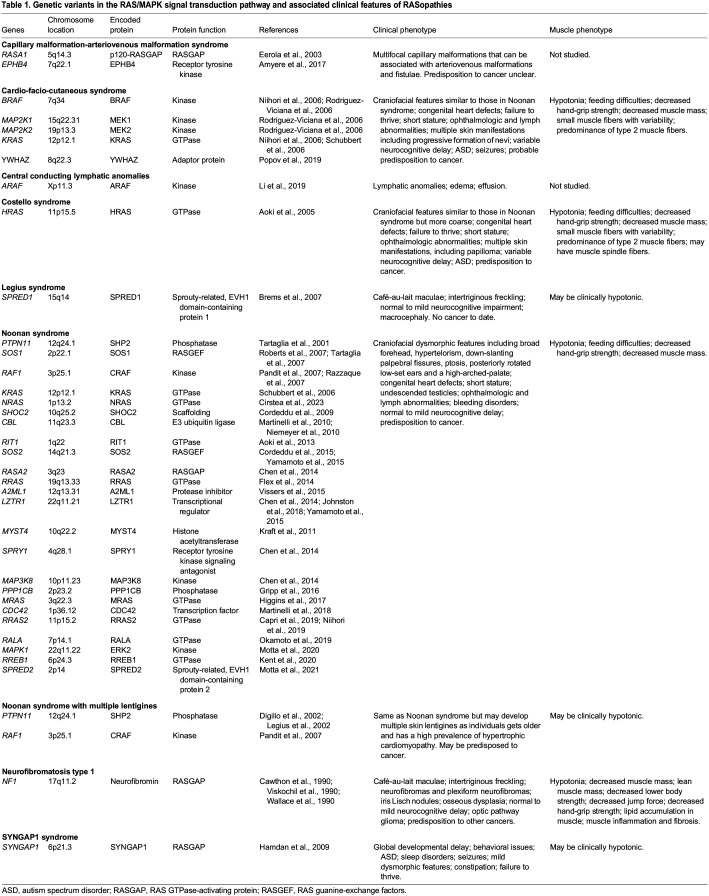
Genetic variants in the RAS/MAPK signal transduction pathway and associated clinical features of RASopathies

RAS is perhaps the best studied signal transducer as it is somatically mutated in approximately 20% of human cancers (Prior et al., 2020). Variants in the canonical RAS genes (*HRAS*, *KRAS* and *NRAS*) often activate RAS signaling, resulting in the dysregulation of the cell cycle and many other vital cellular processes. So, the notion that critical components of the oncogenic RAS pathway can be mutated and activated during development is surprising as its dysregulation during development was thought to be embryonically lethal (Castel et al., 2020). Given this, RASopathies afford us an opportunity to study specific RAS/MAPK pathway-activating gene variants that are present in individuals with a single, germline pathogenic variant. By contrast, in somatic cancers, individuals typically harbor multiple oncogenic variants, not only in RAS, but in many other genes as well. Thus, RASopathies provide us with a unique opportunity to study the role of the RAS/MAPK pathway in normal development and to investigate how its dysregulation impacts specific developmental processes.

**Fig. 2.**
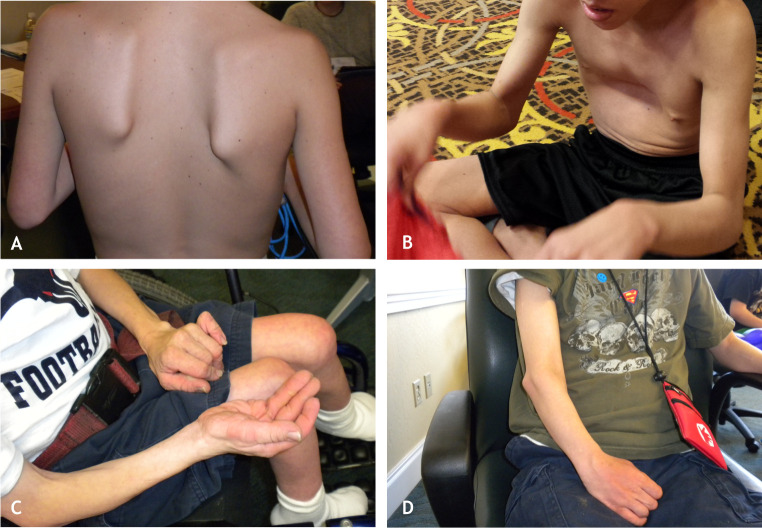
**Reduced muscle mass in RASopathy individuals.** (A) Adolescent male with cardio-facio-cutaneous syndrome (CFC) pictured from behind, who has thin upper arms and uneven scapulae. (B) Another adolescent male with CFC who is pictured from the front and shows overall reduced musculature and an anterior chest deformity. (C) An adult male in his 30s with Costello syndrome (CS). This image demonstrates the paucity of muscle mass on his extremities and shows that reduced musculature persists into adulthood. (D) Another adult male with CS in his 20s showing reduced muscle mass of the upper and lower arm.

In this Review, we focus on how RAS/MAPK dysregulation in RASopathies affects mammalian skeletal muscle development. Many earlier studies into the role of the RAS/MAPK pathway in muscle development were performed in myogenic cell lines (Bennett and Tonks, 1997; Jessen et al., 2013; Scholz et al., 2009). These initial studies suggested that this signaling pathway plays an important role in cell growth and differentiation and showed that it has both positive proliferative effects and negative differentiation effects, depending on the stage of cellular development. More recently, mouse models of RASopathies have provided new insights into the role of the RAS/MAPK signaling pathway in mammalian skeletal muscle development (Andreadi et al., 2012; Brannan et al., 1994; Chen et al., 2009; Kolanczyk et al., 2007; Kossler et al., 2011; Maeda et al., 2021; Sullivan et al., 2014; Summers et al., 2018b; Tidyman et al., 2021), particularly its role in myogenesis (see Glossary, Box 1), and how this process is dysregulated in RASopathies. These models also reveal how the RAS/MAPK pathway interacts with other intracellular signaling pathways in the regulation of this complex developmental process. Our scientific journey studying CS and CFC muscle development began with a very simple clinical observation and question: why are individuals with RASopathies so weak and hypotonic, and why do they present with a striking paucity of muscle mass upon clinical examination (Fig. 2)? We discuss answers to these questions in this Review and explore the potential therapeutic role of RAS pathway inhibition for treating the muscle abnormalities that feature in the clinical presentation of several RASopathies.

**Box 1. Glossary**

**Dynamometry:** the assessment of muscle force from a device used to measure the amount of force skeletal muscle produces.

**Intrafusal fiber:** a specialized muscle fiber that forms the muscle spindle.

**Muscle spindle:** a specialized muscle fiber that is a sensory stretch receptor.

**Myoblast:** a progenitor cell that has the ability to divide but, when it stops dividing, it can develop into a myocyte (muscle cell) in skeletal muscles.

**Myogenesis:** the process of skeletal muscle tissue formation.

**Paraxial mesoderm:** tissue that is in the area of the mesoderm in a developing embryo. The cells give rise to somites, which are discrete areas of tissue flanking both sides of the neural tube. The paraxial mesoderm forms muscle and other tissues in the developing embryo.

**Type 2 muscle fibers:** fast twitch muscle fibers that primarily use the metabolic process of glycolysis. They are important during short, demanding periods of physical activity.

**Satellite cells:** quiescent myogenic cells located between the sarcolemma and basement membrane of mature muscle fibers. In response to injury, they become activated and proliferate, and are responsible for muscle fiber repair and regeneration.

**Western blotting:** a commonly performed protein immunoblotting technique used to detect specific proteins with antibodies directed to specific antigens of the protein. This technique allows one to visualize, distinguish and semi-quantify proteins of interest.

## Genetic variants in the RAS/MAPK pathway that cause RASopathies

RASopathies are caused by germline activating or nullifying pathogenic variants in numerous different genes that encode regulators or other components of the well-studied, yet highly complex, intracellular signaling network that constitutes the RAS/MAPK pathway (Rauen, 2022) (Table 1). RASopathy-associated pathogenic variants in the RAS/MAPK pathway all lead to the intracellular activation or inappropriate signaling of the final effectors of the pathway, namely, ERK1 and ERK2 (also known as MAPK3 and MAPK1, respectively) (Fig. 1). Many RASopathy-associated gene variants are unique, are not found in cancer and activate the RAS/MAPK pathway to a lesser degree, compared to those associated with cancer (Schubbert et al., 2007). As a group, RASopathies have thus shed light on the importance of RAS/MAPK signal transduction and its regulation not only in cancer, but in development as well.

The RAS pathway is highly controlled and important for the transduction of extracellular stimuli to achieve appropriate intracellular responses (Kiel et al., 2021). It is activated by numerous extracellular stimuli via various receptors, including the receptor tyrosine kinases, cytokine receptors, G protein-coupled receptors and extracellular matrix receptors. RAS exists as a GTPase family, of which KRAS, NRAS and HRAS are the best studied. Somatic variants in RAS or in other pathway components activate this vitally important cell signaling network, leading to its dysregulation, which can ultimately lead to cancer (Prior et al., 2020). *KRAS* mutations are the genetic mutations most commonly found in human cancer, followed by mutations in *NRAS* (Papke and Der, 2017), whereas *HRAS* is rarely found to be mutated in human cancer. These genes encode the three canonical RAS proteins and, together with other RAS family members, can cause RASopathies when mutated (Fig. 1). Because all mammalian cells rely on RAS signaling for essential cellular processes, it is understandable that dysregulation of this signaling pathway can impact development in profound ways. The RAS pathway serves as a biochemical signal transduction hub that controls a multitude of downstream effector cascades. The RAS/MAPK effector cascade was the first downstream signaling pathway from RAS to be identified (Fig. 1) (Klomp et al., 2021). Other effector pathways downstream of RAS include: the phosphoinositide 3-kinase (PI3K) pathway, which mediates transcription and anti-apoptotic activities through the AKT protein kinase; RAL guanine nucleotide dissociation stimulator (RALGDS), which is involved in transcription, the cell cycle, cell survival and vesicle transport; the phospholipase Cε (PLCε) cascade, which controls calcium signaling and protein kinase C (PKC) activation; TIAM1, which lies downstream of RAS and regulates RAC1 signaling; and RIN1, which is important for RAS-mediated endocytosis (Klomp et al., 2021).

In its simplest form, RAS in its active state causes the phosphorylation of RAF protein kinases. The RAF protein kinase family includes ARAF, BRAF and CRAF (also known as RAF1). All three of these kinases have been found mutated in different RASopathies: ARAF in CCLAs (Li et al., 2019), BRAF in CFC (Niihori et al., 2006; Rodriguez-Viciana et al., 2006) and CRAF in NS (Pandit et al., 2007; Razzaque et al., 2007). RAF, once activated, then phosphorylates and activates MEK1 (encoded by the gene *MAP2K1*) and MEK2 (encoded by the gene *MAP2K2*). Both *MAP2K1* and *MAP2K2* are mutated in and cause CFC (Rodriguez-Viciana et al., 2006). MEK1 and MEK2 protein isoforms are threonine/tyrosine kinases that share approximately 85% amino acid identity (Wu et al., 1993) but are known to not be redundant in function (Alessandrini et al., 1997; Brott et al., 1993). Both MEK1 and MEK2 can phosphorylate and activate the kinases ERK1 and ERK2. ERK2 mutations have been identified as one of the causes of NS (Motta et al., 2020). Once phosphorylated, ERK1 and ERK2 are the final activated kinases of the RAS/MAPK pathway and phosphorylate their substrates at serine/threonine residues adjacent to a proline consensus sequence (Fig. 1). These sequences are common to other MAPK pathway family members, including the c-Jun N-terminal kinase (JNK) pathway and the p38 pathway (Lavoie et al., 2020). ERK1 and ERK2 phosphorylate hundreds of downstream intracellular and nuclear substrates, which ultimately have varying cellular effects (Unal et al., 2017; Yoon and Seger, 2006). ERK1/ERK2 substrates include nuclear components, transcription factors, membrane proteins and protein kinases that, in turn, control essential cellular functions, including cell cycle progression, differentiation and the control of cellular growth. Once the RAS/MAPK pathway is activated, there are many cellular proteins and feedback loops that negatively regulate the pathway to dampen or inactivate it (Lake et al., 2016). Some of these pathway regulatory components include RAS GTPase-activating proteins (RASGAPs), sprouty (SPRY) proteins, sprouty-related proteins, inhibitor proteins, kinase suppressors and phosphatases (Zaballos et al., 2019).

RASopathies are also caused by pathogenetic mechanisms in both the canonical and non-canonical RAS pathways that lead to a common downstream effect. The different components of these two pathways include receptor tyrosine kinases, GTPases, RASGAPs, RAS guanine-exchange factors (RASGEFs), kinases, scaffolding or adaptor proteins, ubiquitin ligases, phosphatases and pathway inhibitors (Table 1) (Tidyman and Rauen, 2016). Although we have yet to fully understand how the RAS/MAPK pathway functions, it does not operate in a linear fashion, as depicted in its simplest form, but rather as a highly complex network, involving crosstalk and temporal-spatial activation and regulation (Kiel et al., 2021). It is this complexity that underlies the complexity of RASopathy syndromes.

From a pathogenetic perspective, as a disease group, RASopathies are unique as they are pathway based (Fig. 1). Although the genetic variants in the genes that encode the different components of the RAS/MAPK pathway lead to pathogenic changes and pathway dysregulation in diverse ways, the common underlying biochemical outcome shared by all the RASopathies is inappropriate RAS/MAPK pathway activation and pathway dysregulation. This commonality contributes to the overlapping phenotypic features (Table 1) seen among these syndromes and underscores the essential role that the RAS/MAPK pathway fulfills in normal embryonic and postnatal development, as we discuss next.

## RASopathies cause weakness and skeletal muscle abnormalities

One of the primary phenotypic features of the RASopathies is clinical hypotonia, which includes muscle weakness and reduced muscle mass (Fig. 2) (Stevenson et al., 2012). Affected newborns may exhibit hypotonia that is characterized by a lack of muscle tone, muscle weakness, the inability to support their head, impaired motor milestones and delayed neuromuscular development (Table 1). This may result in many infants having feeding difficulties that often require medical and/or surgical intervention, which may result in significant morbidity (Gripp et al., 2019; Pierpont et al., 2014; Romano et al., 2010; Tidyman et al., 2011).

Most studies of the abnormal skeletal muscle features associated with the RASopathies have been performed with individuals with NF1 (Summers et al., 2015). Pediatric individuals with NF1 show a reduced overall cross-sectional muscle size (Stevenson et al., 2005) and reduced lean tissue mass (Dulai et al., 2007) compared to those seen in unaffected control individuals. In addition, pediatric individuals with NF1 have been found to have a significant decrease in lower body strength, compared to that of unaffected age-matched control individuals, as shown by functional tests that included hip-extension dynamometry (Box 1) and peak jumping force measurement (Hockett et al., 2013; Johnson et al., 2012). These individuals also have a marked decrease in forearm muscle strength, as shown by hand-grip dynamometry (Souza et al., 2009). Moreover, in a large study of children with NS, CFC, CS and NF1, they all had a significantly reduced hand-grip strength compared to that of their unaffected sibling control group, with the most profound muscle weakness observed in children with CS and CFC (Stevenson et al., 2012). This study concluded that although muscle strength may progressively increase with age and development, it does not normalize in the RASopathy syndrome groups. Thus, although it was suspected that individuals with RASopathy exhibit weakness upon clinical examination, objective studies have definitively shown that individuals are weaker, and next-step investigations explained why, as described below.

## Skeletal muscle histology in RASopathies

The link between skeletal myopathy and RASopathies first emerged from early case reports on hypotonic infants with CS- or NS-like clinical features. Skeletal muscle biopsies from these patients revealed an increase in the number of intrafusal muscle fibers (Box 1), or muscle spindles (Box 1), and the presence, in most, of small, atrophic, extrafusal muscle fibers (de Boode et al., 1996; Selcen et al., 2001; Stassou et al., 2005; van der Burgt et al., 2007). These histological findings, together with the weakened muscles observed in patients upon clinical examination, led to a more detailed, systematic examination of skeletal muscle in patients with RASopathy, including assessment of muscle biopsies obtained from a cohort of patients aged 11 weeks to 8 years with a molecular diagnosis of CS or CFC (Tidyman et al., 2011). This investigation revealed that most of these biopsies contained excessively small muscle fibers, averaging only 66% of the average standard value for fiber diameter from unaffected age-matched control individuals (Tidyman et al., 2011), and revealed a high degree of variability in myofiber size. Additionally, CS and CFC muscle biopsies were found to have a predominance of type 2 fast muscle fibers (Box 1) with, on average, 20% more type 2 fibers than the standard average value for age in the general population (Johnson et al., 1973). However, muscle biopsies from this CS and CFC cohort did not show an excess of muscle spindles nor, importantly, any evidence of muscle fiber degeneration or regeneration characteristic of dystrophic muscle. In accordance with this finding, muscle biopsies from a cohort of patients with NF1, ranging in age from 3 to 40 years, also showed no signs of muscle degeneration or regeneration, suggesting that the myopathy associated with RASopathies is not degenerative, as can be seen in dystrophies (Summers et al., 2018a). However, in the NF1 cohort, muscle fibrosis and infiltrating mononuclear cells indicative of inflammation were present. Further highlighting the phenotypic variability that is seen among the RASopathy syndromes, there were also high levels of intracellular lipid accumulation in the NF1 muscle biopsies, which was not observed in the CS or CFC biopsies (Summers et al., 2018a; Tidyman et al., 2011). To understand the mechanisms by which dysregulated RAS/MAPK pathway activation produces these skeletal muscle abnormalities in the RASopathies, we need to better understand the biochemical role of the RAS/MAPK pathway in normal skeletal muscle development, as we discuss next.

## Skeletal myogenesis and the RAS/MAPK pathway

**Fig. 3.**
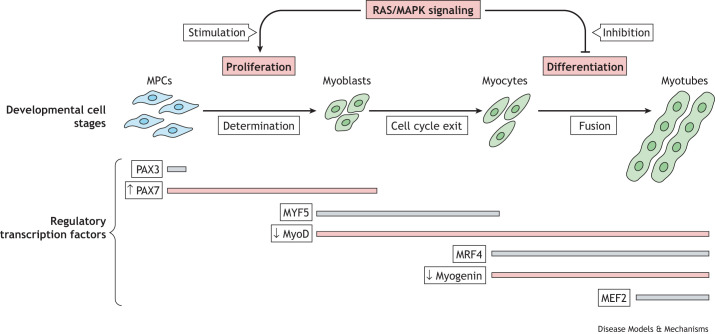
**RAS/MAPK signaling effects on the stages of skeletal myogenesis**. RAS/MAPK pathway signaling stimulates proliferation of muscle precursor cells (MPCs) and myoblasts, and inhibits terminal differentiation of myoblasts and the formation of myotubes. MPCs are irreversibly determined to become proliferating myoblasts. Decreased signaling of the RAS/MAPK pathway induces myoblasts to differentiate, forming post-mitotic muscle cells known as myocytes. Myocytes eventually fuse together to form multinucleated immature muscle fibers, known as myotubes. Shown here are the temporal protein expression patterns of transcription factors that coordinate myogenesis. These transcription factors include PAX3 and PAX7, MyoD, MYF5, myogenin (MYOG), MRF4 and myocyte enhancer factor 2 (MEF2). Current research using mouse models for neurofibromatosis type 1 (NF1), Costello syndrome (CS) and cardio-facio-cutaneous syndrome (CFC) have demonstrated inhibition of myoblast differentiation *in vitro*, as well as a decrease in the number of *MyoD-* and *Myog*-expressing cells, which are markers for differentiation during embryonic skeletal muscle formation. This is indicated by a pink line and a downwards arrow (**↓**) (Kossler et al., 2011; Maeda et al., 2021; Tidyman et al., 2021). Concomitant with this, RASopathy mouse models have demonstrated an increase in the number of cells expressing the PAX7 protein, which is a marker for muscle progenitor cells and myoblast proliferation, as indicated by a pink line and an upwards arrow (**↑**).

Skeletal muscle forms during mammalian embryonic development through a process called myogenesis. This process has been studied using both avian and mammalian model systems as described below. Most skeletal muscle originates from the paraxial mesoderm (Box 1) during embryonic development. This mesodermal tissue segments into discreet units of tissue, called somites, that are adjacent to the neural tube and the notochord (Buckingham et al., 2003). The ventral portion of each somite, the sclerotome, contributes to the formation of cartilage and bone. The dorsal portion, called the dermomyotome, forms the dermis and is the source of muscle precursor cells (MPCs) (Buckingham et al., 2003). As somites mature, cells in the medial region of the dermomyotome elongate and form the first musculature termed the myotome (Gros et al., 2005). MPCs and myocytes from somites at the level of the limbs migrate, following dorsal and ventral paths, to form the limb musculature (Christ and Ordahl, 1995). During their initial migration, and during the development of the muscle masses of the limb, these cells undergo extensive proliferation as the distinct muscles of the limb develop. MPCs subsequently become irreversibly determined as muscle cells called myoblasts (Box 1; Fig. 3) (Tajbakhsh et al., 2009). Myoblasts continue to proliferate before beginning to differentiate to form post-mitotic muscle cells, or myocytes. Myocytes are single nucleated cells that continue their migration and eventually fuse together to form multinucleated, immature muscle fibers, or myotubes. During late embryonic and post-natal development, myotubes mature, cluster and undergo hypertrophy to form mature muscle fibers, or myofibers (Bentzinger et al., 2012). During embryonic development, muscle mass predominantly increases due to the proliferation of myoblasts, whereas during the later stages of development and in the adult, muscle growth is primarily due to muscle fiber hypertrophy (Bentzinger et al., 2012).

The process of myogenesis is controlled by the tightly coordinated, temporal expression of specific transcription factors (Fig. 3). Myogenesis begins in the mesoderm of the somites with the expression of the paired homeobox domain transcription factors, PAX3 and PAX7, which induce the specification of MPCs to the myogenic lineage (Buckingham, 2006; Relaix et al., 2005). The irreversible commitment of MPCs to the myogenic lineage, followed by their differentiation, is orchestrated by the sequential expression of a set of muscle-specific transcription factors, called myogenic regulator factors (MRFs) (Buckingham and Rigby, 2007; Tapscott, 2005). The MRFs consist of four members of the basic helix-loop-helix family of transcription factors, which include MyoD (also known as MYOD1), MYF5, myogenin (MYOG) and MRF4 (also known as MYF6). The cellular presence of MYF5 and MyoD induces the transition of MPCs into myoblasts, causing their cellular reprograming and initiating their differentiation (Cao et al., 2010; Davis et al., 1987). The subsequent expression of the *MYOG* (encoding myogenin) and *MRF4* genes induces myoblasts to transition into non-proliferative myocytes, which fuse and form multinucleated myotubes, which then ultimately mature into muscle fibers (Bentzinger et al., 2012). Another important transcription factor family is myocyte factor 2 (MEF2), consisting of four members encoded by the genes *MEF2A*, *MEF2B*, *MEF2C* and *MEF2D*. *MEF2A* and *MEF2C* are expressed during this stage in skeletal muscle development (Black and Olson, 1998). The MRFs and MEF2 act cooperatively to regulate most muscle-specific gene expression throughout subsequent muscle development and in mature muscle as well (Bentzinger et al., 2012; Black and Olson, 1998; Molkentin and Olson, 1996).

The RAS/MAPK pathway plays an important role in skeletal muscle development, although the extent of its involvement in this process remains to be fully elucidated. Early *in vitro* studies using myoblast cell lines demonstrate that growth factor-induced RAS/MAPK pathway signaling is required for myoblast proliferation, and that a subsequent decrease in RAS pathway activity caused by growth factor withdrawal is required for cell cycle exit and for myoblast differentiation to proceed (Bennett and Tonks, 1997; Tortorella et al., 2001). Likewise, other *in vitro* studies have shown that the overexpression of oncogenic RAS and the constitutively active expression of oncogenic RAF1 and MEK1 protein variants that activate the RAS/MAPK pathway maintain myoblasts in a proliferative state and inhibit myoblast differentiation (Dorman and Johnson, 1999; Konieczny et al., 1989; Olson et al., 1987; Weyman and Wolfman, 1998). These findings suggest that RAS/MAPK signaling plays a central role in controlling MPC and myoblast proliferation and differentiation during embryonic development. In addition to oncogenic variants known to activate the RAS/MAPK pathway, specific syndromic heterozygous gene mutations responsible for RASopathies, including variants in *HRAS* that cause CS and variants in *BRAF*, *MAP2K1* or *MAP2K2* that cause CFC, have also been shown to inhibit myoblast differentiation *in vitro* (Tidyman et al., 2011). Establishing that RASopathy variants dysregulate myogenesis in culture was a key first step to understanding syndrome weakness. However, the use of mouse models was key in distinguishing the mechanisms by which these RASopathy variants impact normal skeletal muscle development, as discussed next.

## RASopathy mouse models – insights into normal and dysregulated muscle development

Most of our mechanistic understanding of the ways in which RASopathies impact normal muscle development has come from mouse models. These models include mouse models of NF1 (Brannan et al., 1994; Kolanczyk et al., 2007; Kossler et al., 2011; Sullivan et al., 2014; Summers et al., 2018b), CFC (Andreadi et al., 2012; Maeda et al., 2021) and CS (Chen et al., 2009; Tidyman et al., 2021), which have been used to investigate the phenotypic characteristics and the molecular etiology of skeletal myopathy, and the effects of RAS/MAPK pathway dysregulation on skeletal muscle development in these RASopathies.

Interestingly, a heterozygous knockout NF1 mouse model (*Nf1*^+/−^) that genocopies the human NF1 syndrome, which is caused by heterozygous mutations in the neurofibromin (*NF1*) gene (Cawthon et al., 1990; Viskochil et al., 1990; Wallace et al., 1990), does not reproduce the muscle phenotype found in individuals with NF1 (Brannan et al., 1994). This heterozygous (*Nf1*^+/−^) mouse model recapitulates very few of the clinical features of NF1, of which impaired bone healing is one. *Nf1*^+/−^ mice have normal muscle size and forelimb grip strength compared to those of wild-type mice and do not exhibit any skeletal muscle anomalies upon histological examination (Kossler et al., 2011; Sullivan et al., 2014). In contrast, the homozygous knockout of *Nf1* in mice is lethal at 12.5-14 days of embryonic development due to cardiac and neural crest defects (Brannan et al., 1994). To circumvent this, two tissue-specific, conditional, homozygous *Nf1* knockout mutants were generated to enable researchers to investigate the effects of NF1 loss on skeletal muscle development. In the first of these mutants, a MyoD-Cre transgene was used to knockout *Nf1* specifically in skeletal muscle (*Nf1^MyoD−/−^*). In the second mutant, *Nf1* was knocked out under the control of a Prx1-Cre transgene (*Nf1^Prx1−/−^*) in a limb mesenchymal cell-specific manner (Kossler et al., 2011; Sullivan et al., 2014). Prx1 is a limb enhancer that drives the expression of Cre recombinase in this transgenic mouse model. In the muscle-specific *Nf1^MyoD−/−^* knockout mouse model, *Nf1* was inactivated in myoblasts at the time of differentiation and remained inactivated during subsequent muscle development (Sullivan et al., 2014). *Nf1^MyoD−/−^* mice were small at birth compared to control littermates but did not exhibit marked skeletal muscle abnormalities (Sullivan et al., 2014). The histological examination of their muscle at 2, 4 and 6 days of age showed no differences in myofiber size relative to that in their wild-type littermates, nor was fibrosis observed in these mice. However, they did show a marked increase in intramyocellular lipid deposits, indicative of profound alterations in lipid metabolism (Sullivan et al., 2014).

In contrast, the *Nf1^Prx1−/−^* mouse model, in which *Nf1* is conditionally knocked out during early development in limb bud mesenchymal cells, survive and exhibit extensive skeletal muscle defects (Kossler et al., 2011). *Nf1^Prx1−/−^* mice show a decrease in body weight of 25%, a 50% reduction in triceps muscle weight and reduced forelimb grip muscle strength, in addition to the accumulation of intramyocellular lipid deposits, relative to those seen in their wild-type littermates (Table 2). Moreover, muscle from *Nf1^Prx1−/−^* mice showed an ∼50% decrease in the total number of muscle fibers per unit area, which was attributed to widespread fibrosis. The coordinated protein expression of the MRF, MyoD and myogenin transcription factors is responsible for myoblast differentiation and their subsequent fusion into multinucleated myotubes; these transcription factors are thus used as molecular markers for myoblast differentiation (Buckingham and Rigby, 2007; Tapscott, 2005). Examination of muscle development between embryonic days 12 and 14 in *Nf1^Prx1−/−^* mice revealed a marked reduction in the number of MyoD- and myogenin-expressing cells in the developing hind limb muscles (Kossler et al., 2011). This was indicative of abnormal myogenesis and of reduced muscle fiber formation during embryonic development, due to the loss of functional NF1 and the resulting inappropriate activation of the RAS/MAPK pathway during this critical period of muscle differentiation. This is the first report of inhibited muscle formation *in vivo* due to the inappropriate activation of the RAS/MAPK pathway in a RASopathy model. In support of this conclusion, satellite cells (Box 1) derived from adult *Nf1^Prx1−/−^* mice also show reduced levels of differentiation *in vitro* compared to wild-type satellite cells (Kossler et al., 2011). Furthermore, the treatment of pregnant females bearing *Nf1^MyoD−/−^* progeny with the MEK inhibitor PD0325901 corrected some myopathic features in 3-day-old mice (Summers et al., 2018b). PD0325901 treatment resulted in increased body weight and decreased muscle lipid accumulation in the *Nf1^MyoD−/−^* pups of treated females compared to those of the untreated controls. By contrast, the treatment of 4-week-old *Nf1^Prx1−/−^* mice with PD0325901 for 8 weeks did not rescue any of their skeletal myopathic features, including muscle mass, strength or muscle lipid accumulation (Summers et al., 2018b).

**Table 2.**
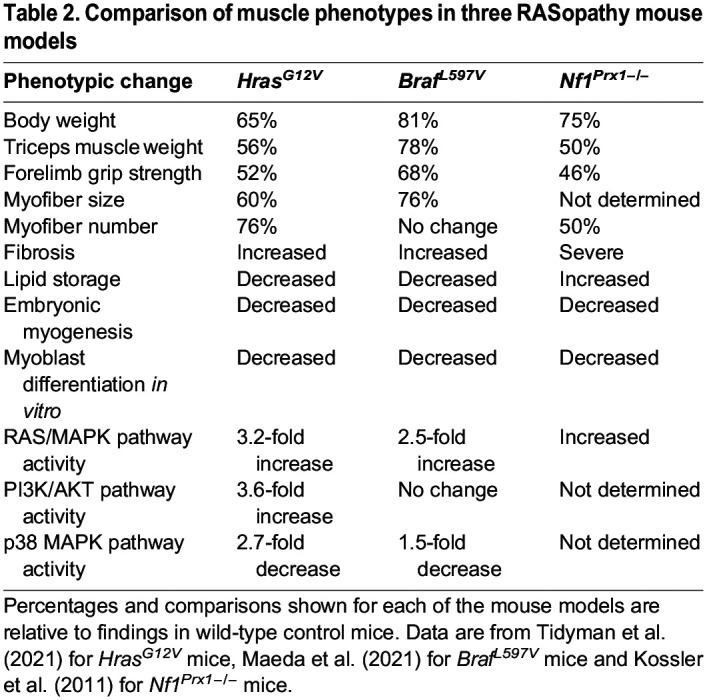
Comparison of muscle phenotypes in three RASopathy mouse models

Two recent studies of the CFC *Braf^L597V^* and the CS *Hras^G12V^* mouse models have yielded significant cellular and molecular information about the role of the RAS/MAPK pathway in normal skeletal muscle development and how the activation of this pathway in RASopathies impacts skeletal muscle development and causes the resulting skeletal myopathy (Maeda et al., 2021; Tidyman et al., 2021). These studies have also shed light on the roles of other intracellular signaling pathways important for skeletal muscle development and their interactions with RAS/MAPK signaling. The CFC *Braf^L597V^* and the CS *Hras^G12V^* mouse models are similar with regards to their effect on skeletal muscle development and the resulting skeletal myopathy; therefore, both models are presented and discussed together. CFC is caused by activating heterozygous germline mutations in the genes *BRAF*, *MAP2K1*, *MAP2K2* or less frequently in *KRAS* (Niihori et al., 2006; Rodriguez-Viciana et al., 2006). The CFC *Braf^L597V^* mouse model (Andreadi et al., 2012) harbors an activating germline heterozygous BRAF p.L597V mutant, which results in an intermediate level of RAS/MAPK pathway activation (Andreadi et al., 2012). Importantly, this variant has been reported in individuals with a clinical diagnosis of CFC and NS and is associated with an overall mild RASopathy phenotype, probably due to the intermediate level of RAS/MAPK pathway activation (Pierpont et al., 2010; Sarkozy et al., 2009). The CFC *Braf^L597V^* mouse model recapitulates numerous clinical characteristics of individuals with CFC, including reduced body size and weight, craniofacial dysmorphia, cardiac abnormalities and skeletal myopathy (Andreadi et al., 2012; Maeda et al., 2021).

CS is caused by heterozygous germline activating variants in *HRAS* (Aoki et al., 2005). The most common missense variant associated with CS is HRAS p.G12S, with p.G12A being the second most common (Rauen, 2007). Although rarer, the HRAS p.G12V missense variant has also been reported in individuals with CS and typically produces a severe phenotype (Quezada and Gripp, 2007). The CS *Hras^G12V^* mouse model harbors an activating heterozygous *Hras^G12V^* mutation and faithfully recapitulates the typical clinical features associated with CS, including decreased birth weight, craniofacial dysmorphia, cardiac abnormalities and a predisposition to cancer (Chen et al., 2009). Both the CS *Hras^G12V^* and CFC *Braf^L597V^* mouse models faithfully reproduce the common pathogenetic etiology of their associated RASopathy syndromes: the activation of the RAS/MAPK pathway. Our studies have shown an approximate 3.2-fold increase in phosphorylated ERK (pERK) levels in skeletal muscle in CS *Hras^G12V^* mice compared to those in wild-type mice, and a 2.5-fold increase in pERK levels in skeletal muscle in CFC *Braf^L597V^* mice compared to those in controls (Table 2) (Maeda et al., 2021; Tidyman et al., 2021). A comparison of RAS/MAPK pathway activation between these two mouse models provides some insights into the role of RAS/MAPK signaling dysregulation on skeletal muscle development and in skeletal myopathy. Both models exhibit reduced body weight and skeletal muscle mass compared to those of wild-type littermates, with CS *Hras^G12V^* mice being more severely affected (Table 2) (Maeda et al., 2021; Tidyman et al., 2021). In CFC *Braf^L597V^* and CS *Hras^G12V^* mice at 1 month of age, the triceps muscle weight is approximately 78% and 56%, respectively, of that of wild-type mice. Likewise, the forelimb grip strength is 68% and 52%, respectively, of that of wild-type mice at 1 month of age (Table 2).

The primary histopathological finding underlying reduced skeletal muscle size and strength in these mutants is a markedly smaller myofiber cross-sectional area in both the CS and CFC mouse models compared to that in wild-type controls (Maeda et al., 2021; Tidyman et al., 2021). In CFC *Braf^L597V^* and CS *Hras^G12V^* mice, the cross-sectional area of gastrocnemius myofibers is 76% and 60%, respectively, of that of age-matched wild-type mice. Interestingly, both slow and fast muscle fiber types have an equivalent reduction in myofiber size in CS and CFC models. One notable difference between the CS *Hras^G12V^* and CFC *Braf^L597V^* mouse models is that there are 76% fewer muscle fibers in the extensor digitorum longus muscle of the lower limb of the CS *Hras^G12V^* mouse compared to those in wild-type controls at 1 month of age, whereas no difference was seen in the CFC *Braf^L597V^* mouse (Table 2). In addition, skeletal muscles from the CS and CFC mice are devoid of lipid deposits compared to those from wild-type mice, which was in marked contrast to the NF1 mouse model, in which lipid accumulation was increased (Table 2) (Kossler et al., 2011).

Similarly to the *Nf1^Prx1−/−^* mouse model, both the CS *Hras^G12V^* and the CFC *Braf^L597V^* mouse models exhibit the inhibition of myogenesis during embryonic development. In both the CS *Hras^G12V^* and the CFC *Braf^L597V^* mice, there is a significant increase in the number of *Pax7*-expressing cells in the developing muscles of the hind limb at embryonic day 14. This indicates an increase in the number of proliferating MPCs and myoblasts compared to those in wild-type mice. Moreover, in both the CS *Hras^G12V^* and the CFC *Braf^L597V^* mice, the embryonic hind limb muscles have significantly fewer *MyoD*- and *Myog*-expressing cells compared to those in wild-type mice (Maeda et al., 2021; Tidyman et al., 2021). As the MyoD and myogenin transcription factors are markers of muscle differentiation, a reduction in their expression indicates that activation of the RAS/MAPK pathway during embryonic development inhibits differentiation and disrupts myogenesis (Table 2). In support of this finding, primary myoblast cells derived from CS *Hras^G12V^* and CFC *Braf^L597V^* neonatal mice show a marked reduction in myosin heavy chain protein levels, which is indicative of myoblast differentiation, and a reduction in myotube formation *in vitro*, compared to myoblasts from wild-type mice (Maeda et al., 2021; Tidyman et al., 2021). This finding also demonstrates that this inhibition of differentiation is intrinsic to CS and CFC myoblast cells and is not dependent on the *in vivo* environment. Importantly, the addition of the MEK inhibitor PD03225901 to CS *Hras^G12V^* and CFC *Braf^L597V^* myoblast cultures rescues this myoblast differentiation defect, resulting in the same time course and degree of myoblast differentiation and myotube formation as seen in wild-type myoblast cultures (Maeda et al., 2021; Tidyman et al., 2021). This finding confirms the role of RAS/MAPK pathway activation as being responsible for the inhibited myogenic differentiation seen in these RASopathy models and highlights an amenable therapeutic route to the correction of the pathway. To further investigate whether RAS/MAPK signaling inhibition *in vivo* could correct RASopathy-associated skeletal myopathy, 3-month-old adult CS *Hras^G12V^* mice were given PD03225901 by oral gavage for 28 days (Tidyman et al., 2021). This treatment rescued the severe myopathic phenotypes observed in CS *Hras^G12V^* mice, including decreased muscle weight, muscle strength and muscle fiber cross-sectional area, to the extent that no statistical differences were recorded in these phenotypes between the treated CS *Hras^G12V^* mice and wild-type controls.

## CS and CFC mouse models reveal that multiple intracellular pathways are dysregulated

Both the CS *Hras^G12V^* and the CFC *Braf^L597V^* mouse models show activation of the RAS/MAPK pathway, as indicated by increased levels of pERK in skeletal muscle (Maeda et al., 2021; Tidyman et al., 2021). The level of RAS/MAPK pathway activation as determined by western blotting (Box 1) in the CS *Hras^G12V^* mouse is 22% higher than in the CFC *Braf^L597V^* mouse (Table 2). In addition to this difference, skeletal muscle from the CS *Hras^G12V^* mouse shows a marked increase in PI3K/AKT pathway activity, as indicated by a 3.6-fold increase of phosphorylated AKT levels compared to those in wild-type muscle, whereas the CFC *Braf^L597V^* mouse does not (Maeda et al., 2021; Tidyman et al., 2021). This difference occurs because HRAS acts as a signaling hub that activates the RAS/MAPK pathway through RAF proteins, and also directly activates the PI3K/AKT pathway. By contrast, BRAF lies downstream of RAS and is not known to directly interact with the PI3K/AKT pathway. PI3K/AKT pathway activation is associated with skeletal muscle hypertrophy and growth due to the activation of mTOR and the stimulation of protein catabolism (Glass, 2010). Inexplicitly, the finding of reduced muscle mass and atrophic myofibers in the CS *Hras^G12V^* mouse is the exact opposite of what would be expected, given the increased levels of PI3K/AKT pathway signaling in this mouse. It is unclear as to why the expected positive effect of an activated PI3K/AKT pathway on muscle growth is not seen in the CS mouse. Instead, the negative effect on muscle growth produced by RAS/MAPK pathway activation appears to be dominant and may override the hypertrophic effect of PI3K/AKT pathway activation, although the underlying mechanism of this effect remains to be explored.

In addition to RAS/MAPK pathway activation, both the CS *Hras^G12V^* and the CFC *Braf^L597V^* mouse models show decreased activity of the p38 MAPK pathway (Table 2) (Maeda et al., 2021; Tidyman et al., 2021). The CS model shows a 2.7-fold decrease and the CFC model shows a 1.5-fold decrease in p38 MAPK pathway activity in mutant skeletal muscle relative to that in wild-type control muscle. It is postulated that crosstalk between the RAS/MAPK and p38 MAPK pathway exists (Aksamitiene et al., 2012). *In vitro* studies in rat myogenic cell lines have shown an inverse correlation between RAS/MAPK and p38 MAPK activity, indicative of mutually inhibitory crosstalk. However, the exact mechanism of this crosstalk remains unknown (Khurana and Dey, 2002). The p38 MAPK pathway is also an essential regulator of skeletal muscle development. The activity of this pathway is necessary for muscle-specific gene expression throughout development and in adult skeletal muscle as well (Keren et al., 2006; Lluis et al., 2006). The p38 MAPK pathway also has an important role in regulating the transcriptional activity of the two primary muscle transcription factors, MyoD and MEF2, which are essential for muscle-specific gene expression (Bentzinger et al., 2012; Black and Olson, 1998; Molkentin et al., 1996). It has been shown that p38 MAPK pathway activity is required for full transcriptional activity of MyoD (Lluis et al., 2005). MyoD dimerizes with its cofactor protein E47 (encoded by *TCF3*) to bind to the promoter region of muscle-specific genes (Tapscott, 2005). Phosphorylation of E47 through the p38 MAPK pathway promotes this dimer formation and facilitates the transcriptional activity of the MyoD/E47 complex (Lluis et al., 2005, 2006). The second major class of muscle transcription factors, MEF2, is also phosphorylated by the p38 MAPK pathway, resulting in the enhancement of its transcriptional activity (Wu et al., 2000; Zhao et al., 1999). Importantly, skeletal muscle from 1-month-old CS *Hras^G12V^* mice show a 2.2-fold decrease in the levels of phosphorylated MEF2C compared to muscle from wild-type mice (Tidyman et al., 2021). In addition, MEF2 has recently been postulated to play a central role in the regulation of muscle hypertrophy. The binding of MEF2 to muscle-specific genes results in an increase in myofiber size. It is proposed that the interaction of MEF2 with the inhibitory muscle regulatory factor MRF4 blocks MEF2 from binding to its target promoters, thereby causing muscle atrophy (Moretti et al., 2016; Schiaffino et al., 2018). It is not known whether MEF2 phosphorylation plays a role in this interaction. However, given that most muscle-specific genes depend on the MyoD and MEF2 transcription factors, a reduction in their activity, due to the RAS/MAPK pathway-mediated inhibition of the p38 MAPK pathway, should have a negative impact on muscle growth (Zetser et al., 1999; Zhao et al., 1999).

## Conclusion

RASopathies provide a unique opportunity to investigate and elucidate the role of RAS/MAPK signaling in mammalian skeletal muscle development and growth, and homeostasis in adult muscle. By studying mouse models of the RASopathies NF1, CS and CFC, researchers have contributed to the identification of a central role for RAS/MAPK signaling in regulating myogenesis, the dysregulation of which results in a novel type of skeletal myopathy, which is characterized by myofiber atrophy and myofiber hypoplasia, in the case of the NF1 and CS mouse models. Myofiber size is a highly regulated process that matches muscle size and strength to functional needs. This process is fundamental to muscle plasticity and homeostasis. The mouse RASopathy models have also revealed that interplay among the RAS/MAPK, PI3K/AKT and p38 MAPK pathways regulates skeletal muscle development and myofiber size. Additionally, as uncovered in the CS *Hras^G12V^* and the CFC *Braf^L597V^* mouse models, RAS/MAPK pathway activation levels and severity of skeletal myopathy also correlate with one another.

There are many key outstanding questions pertaining to skeletal muscle development and RASopathies. Is there any alteration to the population of adult MPCs, known as satellite cells, in RASopathies, given that the RAS/MAPK and p38 MAPK pathways have been shown to be important mediators of their function and activation (Pawlikowski et al., 2017; Segales et al., 2016)? It is also important to determine whether there is a mechanistic connection between an activated RAS pathway observed in RASopathy skeletal muscle and the proclivity of RASopathies to be predisposed to a muscle cancer known as embryonal rhabdomyosarcoma. Embryonal rhabdomyosarcoma (ERMS), also known as PAX fusion-negative rhabdomyosarcoma, is a soft tissue sarcoma that is believed to arise from skeletal muscle progenitor cells (Kashi et al., 2015). Many individuals with RASopathies, including NF1 (Evans et al., 2017), CS (Gripp, 2005) and NS (Kratz et al., 2011), may develop ERMS in childhood. Recent studies have demonstrated that a RAS pathway-activated rhabdomyosarcoma, when challenged with a MEK inhibitor, induced the expression of the transcription factor myogenin and skeletal muscle differentiation (Yohe et al., 2018). This fascinating finding opens the door not only for treatment of RAS pathway-related ERMS in individuals with a RASopathy, but also for a connection between embryonal rhabdomyosarcoma oncogenesis and skeletal myopathy.

Finally, RASopathy mouse models have also provided insights into possible future avenues for treating the skeletal myopathy that features in some RASopathies. For example, the treatment of NF1 *Nf1^Prx1−/−^* mice with a MEK inhibitor *in utero* has been shown to correct the aberrant lipid metabolism that is associated with NF1 (Summers et al., 2018b). In addition, treating adult CS *Hras^G12V^* mice with a MEK inhibitor can fully rescue some of their primary phenotypic abnormalities, including atrophic muscle fibers, muscle size and muscle strength (Tidyman et al., 2021). Such preclinical modeling provides further evidence that MEK inhibitors may provide a robust avenue for treatment in RASopathies and that clinical trials are warranted.
